# Human milk donation in Australia: a qualitative study of donors and potential donors

**DOI:** 10.1186/s13006-025-00782-w

**Published:** 2025-11-18

**Authors:** Claire Newman, Abigail R-A. Edwards, Melissa K. Hyde, Vanessa Clifford, Laura D. Klein, Barbara M. Masser

**Affiliations:** 1 Australian Red Cross Lifeblood, West Melbourne, Australia; 2 School of Psychology, The University of Queensland, Brisbane, QLD Australia; 3 Royal Children’s Hospital, Parkville, Australia; 4 Murdoch Children’s Research Institute, Parkville, Australia; 5 University of Melbourne, Parkville, Australia; 6 Research Unit in Donor Health and Behaviour, Department of Public Health and Primary Care, National Institute for Health and Care Research Blood and Transplant, University of Cambridge, Cambridge, UK

**Keywords:** Donor milk, Milk bank, Human milk donation, Eligibility

## Abstract

**Background:**

Milk banks in Australia rely on the generosity of voluntary donors, enabling them to provide pasteurised donor human milk to vulnerable infants in hospitals. The aim of this study was to explore the experiences and perceptions of donating to a milk bank amongst breastfeeding women who are milk donors, or potentially eligible to donate milk. Having a deeper understanding of the barriers and facilitators for donating to a milk bank will promote sustainability of these services.

**Methods:**

A qualitative descriptive design was used to collect data from 31 participants, either donating, or potentially eligible to donate, to Australian Red Cross Lifeblood’s (Lifeblood) milk bank. Participants were interviewed between May and September 2024 regarding their experiences and perceptions of donation to a milk bank. Interview data were analysed using inductive content analysis.

**Findings:**

Five themes were identified: Knowing about milk donation; being motivated to donate; being (in)eligible to donate; donating needs to be easy and convenient; and trusting a regulated organisation. Many participants initially lacked awareness about milk donation and often learned through online searches or informal sources like social media. They were motivated by a desire to help, a sense of social responsibility, and not wanting to waste milk. Eligibility was influenced by health, lifestyle, and milk supply. Sickness at home was a major source of disruption to collection of milk for donation. Convenience, such as home collection and provision of milk storage bags, facilitated milk donation. Trust in Lifeblood as a regulated organisation played a key role, with many valuing its reputation and safeguards for both donors and recipients.

**Conclusions:**

Breastfeeding women in Australia may be unaware that they can donate their excess milk to a milk bank. Consideration should be given to increasing awareness of milk donation in Australia amongst both breastfeeding women and healthcare workers caring for women who could become milk donors. Further, to improve the recruitment and retention of milk donors, milk banks should prioritise the ease and accessibility of the service to ensure positive donor experiences.

**Supplementary Information:**

The online version contains supplementary material available at 10.1186/s13006-025-00782-w.

## Background

Growing evidence supports the use of donor milk for preterm infants who have insufficient access to maternal milk [1]. Pasteurised donor human milk (PDHM) is recommended as the preferred alternative over infant formula for very preterm infants, based on evidence that it reduces the risk of necrotising enterocolitis [1, 2]. It is estimated that >800,000 infants receive PDHM worldwide each year [3], with demand expected to increase [4]. Considering this increasing need for PDHM, exploring factors that facilitate and/or inhibit women from donating human milk is of value.

International studies and reviews have identified several key facilitators of milk donation. A common motivator is having an oversupply of milk [5, 6], alongside perceiving donation as a solution to wasting excess milk, altruism, the hope of reciprocity if needed, and a belief in the value of providing human milk to vulnerable infants [5–9]. Additional enablers include support from partners and communities [5, 7, 10], as well as knowledge about the donation process, its benefits, and how donated milk is used [5, 7, 11]. Women also report being informed about milk donation by midwives, nurses, lactation specialists and other hospital staff [12–14].

Reported barriers to milk donation can be understood as occurring along a continuum, from initial deterrents to practical challenges faced during the donation process. At the earliest stage, religious or cultural beliefs that do not align with milk donation, as well as misinformation or lack of awareness, may prevent individuals from considering donation altogether [5, 7]. For those who contemplate donating, ineligibility due to medication use, health conditions, or the age of their infant can present significant obstacles [9, 15]. Accessibility issues, such as the absence of nearby milk banks, may also hinder participation [16]. As individuals move closer to engaging in the donation process, further barriers can emerge. Donating often requires a substantial commitment of time and energy to maintain a regular, and sometimes physically and emotionally taxing, expressing routine. Donors have reported the process as time-consuming and physically uncomfortable, citing issues such as sore nipples and breast pain from expressing milk while also breastfeeding [5, 7, 11]. Logistical difficulties further complicate the process and may include obtaining serology screening, storing large volumes of frozen milk, and traveling to donation sites [6, 7, 13].

While international research has identified common barriers and facilitators to milk donation, there is a notable lack of evidence specific to the Australian context. Cultural norms, healthcare systems, and milk bank infrastructure vary significantly across countries, meaning that findings from overseas may not fully capture the experiences or challenges faced by Australian donors. Although some Australian studies have explored the experiences of ineligible donors in New South Wales and South Australia [9], and bereaved milk donors [17, 18], there is a clear gap in research examining the perspectives of donors and potential donors more broadly. Understanding these experiences within the Australian context is essential for identifying context-specific barriers and enablers, and for informing strategies to improve donor recruitment and retention in local milk banks.

## Methods

### Aims

This study aimed to explore the experiences and perceptions of breastfeeding individuals regarding milk donation to Lifeblood’s milk bank, including those who had recently donated milk to Lifeblood (donors) and those who were potentially eligible to donate (potential donors).

### Design

The study used a qualitative descriptive design to provide a comprehensive description of the factors facilitating or inhibiting milk donation. Theoretically underpinned by naturalism, qualitative description aims to describe, rather than explain, how individuals perceive and experience phenomena [19]. We selected qualitative description as our approach to address the research aim, given that we sought to capture and summarise participants’ experiences, rather than explain them (as per more interpretative methodologies such as phenomenology or grounded theory). Data are typically collected through interviews or focus groups and then subject to qualitative content analysis [20]. Analysis in qualitative description is characterised by an approach that stays close to the data to provide a rich description of the phenomena using the participant’s own words and meanings [20].

### Setting

Lifeblood is the national blood service in Australia. Lifeblood’s milk bank began recruiting donors in July 2018 and providing PDHM to hospitals in September 2018. Lifeblood is now the primary supplier of PDHM in Australia and around 1,800 women have donated approximately 24,000 L of milk to Lifeblood since the service began [21]. Donation is voluntary and non-remunerated. To donate, women must undergo eligibility screening, infectious diseases blood testing and have at least 3 L of surplus breast milk collected within the past 10 weeks, stored according to Lifeblood guidelines. At the time of data collection, Lifeblood recruited milk donors from metropolitan Sydney (New South Wales), Brisbane (Queensland) and Adelaide (South Australia). Further detail regarding the requirements for Lifeblood milk donors and details of Lifeblood-specific milk banking processes have been published elsewhere [21, 22].

### Participant recruitment

The sample included 16 donors and 15 potential donors. Participants were purposively selected based on their relevant experience with expressing and storing breastmilk, consideration of milk donation, being recruited to and donating to a milk bank (donors only), or being potentially eligible to donate to a milk bank (potential donors only). Participants were recruited between May and September 2024. Donors were recruited via a flyer provided to them during a routine home visit by milk bank staff. All women who donated milk to Lifeblood during the recruitment period were eligible to participate with the exception of bereaved donors who were excluded, as Australian bereaved donors generally have unique motivations and experiences of donating milk [17]. Potential donors were recruited via a social media advertisement, from which they accessed a screening questionnaire to determine their eligibility to participate. Potential donors were eligible if they were expressing and storing milk for an infant under 12-months of age and living in a postcode where Lifeblood collects milk but were not donating to any milk bank. Respondents were offered a gift-voucher (AUD 80) as an appreciation of their time to participate in the interview.

### Data collection

Semi-structured interviews were undertaken by authors CN and AE via telephone using a semi-structured interview guide which was derived following consultation with key stakeholders (see Additional File 1). Interview topics focused on participants’ perceptions and experiences of milk donation including motivations, uses of donor milk, expressing milk, and factors that inhibited or facilitated donation. Interviews ranged in duration from 15 to 47 min. Interviews were audio-recorded and transcribed to create a text for analysis.

### Data analysis

Data were analysed thematically using an inductive approach consistent with Elo and Kyngäs [23] qualitative content analysis process. CN and AE immersed themselves in the data through reading and re-reading and then independently applied open-coded notes using NVivo, v.14 [24] to describe content. Through an iterative process, codes were categorised and themes generated. To help minimise researcher bias, several strategies were employed, including the incorporation of participants’ own words and ongoing discussions within the research team to reflect on emerging interpretations of the data with a conscious acknowledgement of the potential influences of researchers’ personal beliefs, assumptions and prior experiences. Consensus regarding the final themes and subthemes was achieved through discussion with the research team. The final themes were inclusive of both donor and potential donor experiences and perceptions. Barriers and facilitators for milk donation were derived from qualitative themes and summarised in a table.

### Ethics approval and consent to participate

Ethical approval to conduct the study was received from Lifeblood Human Research Ethics Committee (2024#18-LNR) and the University of Queensland Human Research Ethics Committee (2024/HE000885). Participants were provided with a study information sheet and signed an electronic consent form prior to participation in an interview.

### Findings

Participant demographic data is provided in Table 1. Most participants resided in New South Wales (*n* = 14, 45%), were aged between 30 and 39 years old (*n* = 17, 55%) and had one or two children (*n* = 16, 52% and *n* = 10, 32%, respectively). Most participants were married (*n* = 24, 78%) and almost half were tertiary degree educated (*n* = 15, 48%). Most participants did not consider themselves to belong to an ethnic minority group in Australia (*n* = 27, 87%). Three potential donors (20%) and two (13%) donors had previously given milk directly to other families (peer-sharing).

Five themes were identified: knowing about milk donation; being motivated to donate; being (in)eligible to donate; donating needs to be easy and convenient; and trusting a regulated organisation. The themes and subthemes derived from analysis are provided in Table 2.

**Table 1:** Participant demographic data

Descriptor	Donor	Potential donor	Total	
	*n*	*n*	*n* (%)	
**State**				
New South Wales	7	7	14 (45)	
Queensland	6	5	11 (36)	
South Australia	3	3	6 (19)	
**Age (years)**				
18–24	5	3	8 (26)	
30–39	8	9	17 (55)	
40+	3	3	6 (19)	
**Number of children**				
1	10	6	16 (52)	
2	4	6	10 (32)	
3	1	3	4 (13)	
4	1	0	1 (3)	
**Marital status**				
Married	13	11	24 (78)	
De-facto	3	3	6 (19)	
Separated/Divorced	0	1	1 (3)	
**Highest level of education**				
College certificate/ diploma	3	2	5 (16)	
Degree/tertiary qualification	8	7	15 (48)	
Postgraduate degree	5	6	11 (36)	
Ethnic minority*				
No	14	13	27 (87)	
Yes	2	2	4 (13)	

*Racial, ethnic or ethnic-religious group which is a minority in Australian society

**Table 2:** Themes and subthemes

Theme	Sub-theme	
Knowing about milk donation	I don’t know enough about donating milk	
	Learning about milk donation	
Being motivated to donate	Wanting to help	
	Feeling social responsibility	
	Valuing milk as a resource that shouldn’t be wasted	
Being (in)eligible to donate	Being screened for eligibility to donate	
	Needing an excess supply of milk to donate	
	Being deferred due to sickness	
Donating needs to be easy and convenient	Having a straightforward, easy and convenient process	
	Requiring storage space	
Trusting a regulated organisation	Trusting the formal procedures of a milk bank	
	Choosing Lifeblood because of their reputation	

## Knowing about milk donation

### I don’t know enough about milk donation

Both donors (D) and potential donors (PD) spoke about not knowing, or previously not knowing, what milk donation was. For potential donors, having limited or no knowledge about milk donation inhibited their ability to donate and they talked about having ‘no idea’ or ‘no knowledge’ and ‘not knowing anything’ about milk donation, or having never given the topic previous thought or serious consideration: “I genuinely haven’t really thought about it too much” (PD2). One potential donor described how this lack of knowledge about milk donation was justified by them not knowing if there was a need for donated milk:

The thought (of donating) has crossed my mind… (but) not knowing if there’s much of a need, I haven’t really investigated it further (PD8)

For donors, this lack of awareness led them to reflect on missed opportunities to donate with previous infants:

I didn’t realise it was a thing until after my third (baby) and I felt a bit bad because I thought well, I probably could have expressed with the other two (D10)

Potential donors wanted to know more about various aspects of milk donation, specifically: eligibility criteria; the minimum volume of milk required to donate, how milk needs to be expressed and stored, and how expressed milk is collected for donation or where it needs to be taken:

I think if I was more aware of the process that would be of benefit to me because then I would be able to know whether I could do it or what it would require and I would be able to think it through a little bit more (PD1)

### Learning about milk donation

Most donors, and potential donors who were aware of milk donation, talked about doing ‘self-research’ online to find out more information. Using Google and being directed to the Lifeblood website was common:

I looked up (online) just the idea of donating… just to get an idea of what donation would look like in terms of how much I could donate at a time and how exactly it’s used, and the milk bank provided a lot of other information…There was a lot of that information on the website (PD15)

Participants spoke about how they became aware of milk donation. One donor learned about milk donation when her premature baby received PDHM. For other donors and potential donors, their awareness of milk donation came from viewing posters at Lifeblood blood donor centres, social media posts, pamphlets on maternity wards, and advertisements for Lifeblood’s milk bank on television or social media:

Ads pop up on my Facebook saying, ‘you know that you can donate milk with the Red Cross?’ So, I learned about that, so I registered and became a milk donor like that (D6)

Many donors and potential donors felt that women should be learning about milk donation from healthcare professionals, although only two donors spoke of being informed about milk donation in this way. One donor was provided with information about milk donation by nurses in a post-natal hospital unit when she requested it. Two donors had discussions with their GP; one initiated by a donor where their GP did not know about milk donation, and the other initiated by a GP in response to the donor’s oversupply of milk:

I mentioned to my GP that I was donating milk, and she had never heard of it either…. she had no idea that breastmilk donation was a thing in Australia (D3)

Two donors highlighted that if they had a conversation about milk donation with a healthcare professional, a trustworthy source, they may have considered donating milk sooner than they did:

Maybe in hospital, if someone had spoken to me, like, ‘Hey, you’ve clearly got pretty good milk supply,’… but I guess if someone had mentioned it there, I probably would’ve thought about it a lot earlier (D11)

Despite the absence of conversations with healthcare professionals about milk donation, participants expressed a desire to receive information about milk donation from GPs, doctors, midwives, and child and family health nurses. Participants felt that being provided with some ‘general’ and ‘simple’ information about milk donation, ‘without putting pressure on’, would be ‘helpful’ to inform families that it was ‘an option’. Participants talked about opportunities where such information could be provided by healthcare professionals, including during hospital and community-based pre-and post-natal appointments.

## Being motivated to donate

### Wanting to help

Most participants talked about a desire to ‘help’ as a motivator for donating milk, either wanting to help others in general or babies specifically:

I just love the idea that I can help somebody with the excess that I have (D6)

Once you’ve got a baby of your own, you really want to help anyone else’s baby, because they’re just so helpless (D3)

For one donor, being told how much they had donated and what hospitals their milk had gone to solidified their understanding that through milk donation they were helping babies. This provided continued motivation for them to donate:

Each time they’ve come to collect, they’ve let me know where my milk has gone, and how much I have actually donated. That gives you a lot more motivation to keep on pumping, and keep on providing, because you know that it’s helping babies (D3)

Other donors and potential donors were motivated by an acute awareness of the need for donor milk for premature babies, either from personal or professional experience:

I think seeing all the really premmie babies and babies with health conditions and things, I think that pulls on your heartstrings a bit and you’re like, ‘I want to help those babies if I can’ (D4)

### Feeling socially responsible

Some donors and potential donors spoke about feeling that they should donate milk, simply because they were able to do so. They felt ‘fortunate’, ‘glad’ and ‘grateful’ that not only were they able to breastfeed their own baby, but they produced enough milk to be able to help feed someone else’s baby too:

I’m producing milk already; if I can produce a little bit more and help someone else, why wouldn’t I? (PD15)

Donors and potential donors also talked about being motivated by the anticipation of reciprocity – that iqs if they ever needed to access donor milk that it would be available to them. There was acknowledgement that a reciprocation of social responsibility was required, where they hoped others would donate their milk for them if needed:

I would like to think that if I had a baby in the NICU, that I would have access to donor milk if I needed it as well. So, it’s a little bit of karma going around, so I’ll do something for someone else in the hope that one day I don’t need it but if I did, that someone else would do the same for me (PD1)

### Valuing milk as a resource not to be wasted

Donors and potential donors talked about their milk being a ‘valuable’ resource, one that they had to ‘work’ and ‘take time’ to express. These participants felt motivated to donate milk so that this valuable resource was not wasted:

My baby didn’t need it, I’d rather it go to another baby that does need it, than just sit there and get wasted (D4)

One donor felt comforted by the fact that even if her donated milk was unable to be given to babies, it could still be used for research and would not be wasted:

It was comforting as well, to know that even if for whatever reason they couldn’t give it to babies, like if they tested it and if there was something wrong with the milk or for whatever reason, that it wouldn’t go to waste. It was actually going to research purposes (D2)

Other donors and non-donors spoke of their need to simply empty their freezers of expressed milk they had stored, and donation provided them an opportunity to do so and avoid waste:

Then the freezer got taken over and I was like, okay, what am I going to do with all this? I don’t need it. And so that’s when I looked into donation (D12)

## Being (in)eligible to donate

### Being screened for eligibility to donate

Prior to donation, prospective donors undergo screening to determine if they are eligible to donate. Some donors spoke of already having a lifestyle or medical history compatible with being eligible, and/or being mindful of what they consume to remain eligible to donate milk. Whereas some potential donors talked about being ineligible to donate due to medications they were taking, their lifestyle, or because of their caffeine intake:

They require that I don’t have any illness, any medications, maximum two coffees a day, no alcohol, no smoking. Those kind of things (are) consistent with my lifestyle (D6)

I drink coffee, so if it was – if there was a requirement that you didn’t have to drink coffee, that would be a bit of a barrier for me, personally. And because I personally feel comfortable… consuming caffeine whilst breastfeeding (PD7)

Some donors expressed that it would be helpful to receive clear and consistent information about eligibility criteria early. Often, women commence the screening process after they have already expressed and stored milk in excess of their own infant’s needs. Whilst Lifeblood accepts milk that has been expressed and stored prior to initial screening, some of this milk may not meet the criteria for donation due to factors such as caffeine intake, medication use, or improper storage or collection methods. If donors were unable to recall the specific dates when these factors may have affected the milk, the entire stored supply was deemed ineligible.

The one challenging thing was remembering exact dates of any medications. I’m quite aware of what I can take to feed if I’m feeding (my baby), but because they were a lot more strict on different things for donating to premmie babies, there were times that I’d had to take Ibuprofen and that was okay for (my baby) but they didn’t want that for the milk for the donation. So, trying to remember exact dates as to when I’d done that was probably the biggest challenge (D13)

### Needing an excess supply of milk to donate

Donors talked about expressing more milk than was being consumed by their baby. This excess of milk enabled them to donate. A few potential donors also talked about having an oversupply of milk or milk stored that they considered excess to their baby’s needs. However, most potential donors described not having any, or enough, excess milk to be able to become a milk donor:

I just had such a massive oversupply of milk… So, there’s a lot going into the freezer…I can keep some supply for myself that I already have, and then donate the excess (D14)

Whatever I got was exactly what (my baby) took and I thought about wanting to donate but I never had extra (PD14)

Some donors expressed, or expressed additional, milk for the sole purpose of donation. Whilst these donors did not report any ill-effects from expressing extra milk to donate, some potential donors expressed concerns about the risks associated with purposely inducing an oversupply of milk:

I just started building up my supply so I had enough milk for my toddler of whatever I wanted to give him, plus one feed a day for (donation) (D8)

I started (expressing) for the purpose of donation (D11)

(I would need to) purposely start doing extra pumps for the intention just to donate… I’d be concerned about bringing in too much and getting mastitis… it’s kind of a double-edged sword, really. You might want to donate, but it’s difficult to do it (PD4)

Another aspect of needing excess milk to donate talked about by both donors and potential donors was Lifeblood’s requirement for donors to have a minimum of 3 litres of milk less than 10 weeks old to donate. Some potential donors talked about feeling ‘daunted’ and ‘overwhelmed’ at the thought of needing to ‘stockpile’ 3 litres of milk for donation. This minimum amount was described as ‘too much’ and ‘difficult’. It was an ‘obstacle’ and ‘barrier’ to them becoming milk donors as it simply wasn’t ‘achievable’:

The thing that interrupted me a little bit was the amount that I would have to have extra to be eligible to donate and I was doing the numbers and everything, going ‘what is it? An extra 300 [mls] per week’ and going ‘I don’t know if I quite fit that bill’…There’s probably a good reason why there’s that minimum amount, but that, for me, has been a bit of an obstacle (PD14)

### Being deferred due to sickness

Half of the donors interviewed experienced being unable to donate their milk due to sickness. Some experienced temporarily deferrals when they themselves were unwell. Sometimes deferral periods were lengthy as they included time before and after symptoms where milk could not be accepted for donation. Data were collected during winter where donors spoke of having consecutive deferral periods due to sickness which resulted in prolonged times where they were unable to donate milk:

We just had lots of colds at the beginning, like we were sick about four times in a row…. we got COVID and that got a long deferral period, then we got (a) cold somewhere, then another cold and then I think another one. It was crazy…. it was very long, like six weeks of just being sick (D8)

The impact of infant/child sickness on delaying or cancelling milk collection was also talked about by donors. For those who had children in daycare, these periods were frequent:

The one challenge that I had was them not being able to come to collect if we had anyone sick in the house, with two small children generally someone in the household is sick… We had to all be healthy for two weeks which with small kids is basically impossible in winter (D13)

Not fully understanding the rules around sickness-related deferrals, and disruptions to milk collection associated with family sickness, was highlighted by some donors. Some donors described how during a call with the donor coordinator they realised that they either couldn’t donate or no longer had the minimum amount needed for donation:

So that’s a bit limiting, especially if you don’t know about it before you call because sort of you are storing, storing and then you call, “Hi guys, I’ve got 4 litres” and then you get asked all the questions and you realise, “Oh okay, I don’t have 4 litres because I can’t give you half of what I already have (D8)

## Donating needs to be easy and convenient

### Wanting a straightforward, easy and convenient process

Donors spoke of how the convenience of the process enabled them to donate. They described their experiences of recruitment, screening and milk collections as ‘easy’, ‘straightforward’, ‘convenient’, and ‘simple’. Donors described how they appreciated the time and hassle it saved them by having milk bank staff come to their home to collect both the required blood sample and their milk for donation. Similarly, potential donors felt that milk donation would be easier for them to do with a home collection service. One donor described how the convenience of this home-based service influenced their decision to donate to Lifeblood over other milk banks:

(with an alternative milk bank) I’d have to go see the GP for a blood test, and I just don’t think that was something that was doable quickly at that point in time. And it was just a lot more hassle…. I also had to donate in the city, drop-off, which is not an easy drive with a young baby…. And then I got into the Lifeblood one, and I was like, ‘This is a lot easier!’ (D7)

Donors also described their experiences of the milk donation process being ‘quick’ and ‘efficient’ which supported them to ensure they didn’t waste milk. As their milk was collected soon after calling, their freezer didn’t get too full of milk waiting to be donated or wasn’t left to go beyond the eligible post-expression timeframe:

I think that it was just very efficient in how quickly their response time was, and setting up for collection, because obviously, if it takes a long time, then a lot of the milk is going to waste (D14)

The milk collection process was also described as being positively facilitated by donor coordinators, who were described as ‘easy to contact’ and ‘flexible’. Donors spoke of feeling enabled and encouraged by having pleasant interactions with the donor coordinators. Contact was facilitated by having the direct numbers of the donor coordinators to arrange appointments:

They’re all so kind… for me, expressing milk is such a small part. I know it’s not (a) small part, but for me it’s like a very small thing… (but they) praise me that, ‘Oh, you did a really great job’, like this. So, all their kind words makes (donating) real easy (D16)

They said just to send them a text message or give them a call… it’s nice having I guess a bit of continuity, knowing that I had a great experience with the two people I spoke with the second time whose mobile numbers I have (D11)

Although potential donors had no experience of the process of donating to a milk bank, some talked about being willing to donate if the process was not too difficult, complicated and/or time-consuming. Some potential donors felt that donating milk would require time and effort they simply did not have to give. Others talked about needing ‘the mental capacity’, ‘personal resources’, and ‘a calm state of mind’ to donate milk, and if these were not possible, they felt unable to donate:

But we’re already sometimes, as you can imagine with two young kids, stretched to the limit. And so yes, it would just be yet another thing to try and do (PD8)

If it (is) super complicated to donate to a milk bank that’s just effort and stuff on top of what I’m already doing (PD13)

### Requiring storage space and equipment

Donors discussed already having the necessary equipment to express milk prior to their decision to donate. Others noted that the biggest expense associated with milk donation were milk bags to store expressed milk, and donors appreciated that these were provided by the milk bank:

I suppose cost of living is going up…So, I might not have continued to donate as much for as long if I had to keep on buying my own bags (D3)

Similarly, some potential donors felt women would be encouraged to donate if they were provided with milk bags and other equipment such as breast pumps:

Milk expressing bags, and things like that, they’re quite expensive – I’m not sure how it works, but if that was supplied to the donor, or even the facilities to pump, or use a pump, when required, or to hire one for free, or something like that, that would definitely be an encouragement, I guess it would make it easier for someone to access and to donate (PD3)

Both donors and potential donors talked about the need to have freezer storage space to facilitate being a milk donor, with some donors challenged by lack of storage space:

Our freezer has literally got no space in it, at all. There’s no space for anything. It’s just milk in our freezer pretty much at the moment. It’s a practical problem, as much as I’m laughing about it, it’s actually an issue (D12)

## Trusting a regulated organisation

### Trusting the formal procedures of a milk bank

Both donors and potential donors talked about trusting the processes that the Lifeblood milk bank has to ‘safeguard’ the recipients of donor milk. Some participants also talked of feeling protected as a donor or prospective donor by the safeguards in place and felt reassured that they would not be responsible if a recipient experienced adverse effects from donor milk:

Just the quality control of something like Lifeblood, the extra testing that they do to make sure the milk is safe and not contaminated in any way. I guess you’d just want to make sure that you’re not kind of inadvertently transferring any bacteria or illnesses to another baby. Like that would just be kind of horrible if that happened. So yeah, I guess that little added extra layer of protection is probably a bit more reassuring (D2)

I think I probably would prefer (to donate to) the milk bank even for similar reasons as things like organ donation, they keep donors private for reasons such as asking for compensation or different high conflicts that could evolve when you know where you’re getting a donated, I guess, body part from. And I can anticipate there’d be issues, for example, some people have (a dairy) allergy and if I donated milk which had dairy in it and someone’s baby had a reaction and I know them they’d get personally angry at me. (PD5)

Some participants also emphasised the absence of safeguards if they shared their milk informally (e.g., peer-to-peer), with donating to a milk bank being the ‘safer’ option. Some potential donors preferred the idea of donating to a milk bank over informally sharing their milk because they felt their milk would be handled and stored with care. They also trusted that Lifeblood would do the right thing with their milk by ensuring it went to babies who needed it, releasing them from such responsibility:

I would prefer to give it to a milk bank, because I know that they would handle it appropriately, store it appropriately, and give it to the babies that need it the most. So, I don’t need to think about any of those things (PD10)

### Choosing lifeblood because of their reputation

Lifeblood was described as a ‘respected’ and ‘legitimate’ organisation that people associate with blood donation. One donor described how these factors influenced her decision around where to donate:

I was more interested in donating to Lifeblood than I was to the other places that I found online… because you guys have a reputation of blood donation, so when it’s like a big company that you already know about, and you hear the work that they’re doing. Everyone knows about donating blood, but I didn’t know about the donating of milk. So, I thought, oh, I already know this company does so much for so many people, then why wouldn’t I try going through them, when I hadn’t heard of the other company at all? (D3)

Six donors and five potential donors had previously donated blood and were, therefore, familiar with Lifeblood. One donor described how having a previous and positive relationship with Lifeblood as a blood donor influenced her decision to donate milk:

I mean I always have a really fantastic experience going to the donor centres to donate plasma… the staff have always made me feel really comfortable and over the years you kind of get to know people as well. It’s quite a nice experience going into the centres. Yeah, I thought donating breastmilk is probably a bit of a different process, but I kind of expected the staff to be the same level of friendliness and openness. And to get that support as well. (D2)

The barriers and facilitators to milk donation, identified from the qualitative themes, are presented in Table 3.

## Discussion

Barriers and facilitators to milk donation experienced or perceived by participants related to their knowledge/awareness of donation, motivations such as helping others and having an oversupply, (in)eligibility to donate, ease and accessibility of milk banking services, and having a milk service delivered by a trusted and reputable organisation.

All participants expressed motivations for donating their milk which aligned with findings from previous research [5–7, 12, 13]. However, this study also highlighted that a lack of awareness about milk banks and the donation process may be a significant barrier to donor recruitment. This finding mirrors patterns observed in other forms of donation, such as blood and organ donation, where awareness has been shown to be a key predictor of registration and participation [25, 26]. Regarding milk donation, a recent study in China found that insufficient knowledge about human milk banks was a barrier among women who had not donated [27]. Donors in the current study also expressed a desire to receive information about donation requirements earlier from health professionals, to help ensure proper milk collection and storage. Providing timely and accessible information about milk donation, including transparent guidance, may therefore support women in making informed decisions and potentially enable earlier or increased participation [16]. Further research is needed to examine how awareness, or the lack thereof, affects the pool of potential milk donors across Australia.

**Table 3:** Barriers and facilitators for milk donation derived from themes

Influencing factor	Barrier to donation	Facilitator of donation
Knowing about milk donation	Not knowing how to donate milk	Having an awareness of milk donation
	Not knowing enough about milk donation to decide if it is something they want or are able to do	Receiving information about donation from a trusted source (e.g. a healthcare professional)
		Being able to access information online
Being motivated to donate		Wanting to help others
	Not understanding need for donor milk	Understanding need for donor milk
		Providing alternative to wasting excess milk
		Receiving feedback that milk donated is directly helping babies
Being (in)eligible to donate	Not having enough or excess milk	Having excess supply of milk
	Contraindicatory medical conditions	Compatible with health and lifestyle
	Taking medications that are incompatible with donation	Being able to express extra milk for donation
	Deferrals due to sickness (of donor or in household)	
Donating needs to be easy and convenient	Lack of personal capacity (time, headspace)	Easy recruitment/screening process
	Donation information and materials only available in English	Positive, supportive interactions with milk bank staff
		No cost to donor (i.e. supplied with milk storage bags)
Trusting a regulated industry		Reassured by milk processes for testing and dispensing milk
		Safeguards to protect donor’s anonymity
		Lifeblood’ as reputable and trusted brand

Interestingly, healthcare professionals were not often reported as a source of information about milk donation for participants in this study, unlike in other studies where women in France and the US reported being informed about milk donation by a range of healthcare professionals involved in ante- and post-natal care [13, 14]. The limited engagement of healthcare professionals with women regarding milk donation may reflect the relatively recent development and expansion of milk banking services in Australia, especially when compared to countries like the United States. As such, the evolving role of Australian healthcare professionals in raising awareness and providing information about milk donation presents an important area for further exploration.

The study findings provide further evidence that processes for milk donation need to be easy and convenient for donors. Our findings in this regard are similar to those reported in an earlier Australian study; when asked about the potential for a milk bank in South Australia, breastfeeding mothers unanimously supported this idea, provided the process for donation was convenient and safe [28]. Milk banks that provide a service that is accessible, convenient, supportive and empowering are more likely to attract, engage and retain donors [8]. In the current study, potential donors expressed hesitation when the process appeared time-consuming, complicated, or required additional effort. Similarly, milk bank processes that are time and energy consuming have been identified as challenges experienced by milk donors [11] and factors that discourage potential donors [28]. The availability of equipment, such as milk storage bags and adequate freezer space, also influenced donors’ ability to donate; highlighting that some women may experience financial barriers to donation. It is further acknowledged that challenges associated with donating may stem from systemic barriers within the milk service, such as communication, infrastructure, and technical issues, which were not explored in this study. Systemic barriers have been associated with negative milk donor experiences [5, 7] and warrant further consideration in the Australian context.

Donors highlighted that their relationships with milk bank staff were an important part of their donation experience. Milk bank staff are recognised as a source of support and information [5, 7, 29]. Participants identified that milk bank staff provided information related to their eligibility to donate as well as milk expression, collection and storage. The depth and transparency of information provided to milk donors has been identified as an important component of their experience, with a constant relationship and dialogue between staff and donor required to ensure the safety of donated milk [30]. Having appropriately trained milk bank staff has been highlighted to be of utmost importance [30]. Although not reported by donors in the current study, negative interactions with milk bank staff, such as a lack of friendliness, appreciation, or flexibility, have been identified in previous research as barriers to milk donation [11]. While donors consistently reported positive experiences with milk bank staff, enhancing organisational support for staff education is encouraged to help sustain positive and supportive relationships with both donors and potential donors. Further research on the support needs of milk bank staff to ensure their wellbeing when working with potentially traumatised donors, such as those who have suffered infant loss, is also needed.

Finally, study findings highlighted the influence of collection agency reputation as a factor that impacts milk donation in Australia. Participants spoke of choosing to donate to a trusted organisation, with a brand associated with the national blood service. Reputational trust may be explained by the shared safety and quality assurance standards the national blood service is known for, including standards related to processing, manufacturing and donor management [31]. Having an organisation responsible for both blood and milk donation is relatively uncommon, with a few similar models existing in Switzerland, Canada, the US and Germany (see for example: [31, 32]). Whilst the impact of organisational reputation is recognised to influence blood donation [33–35], it is an under-researched phenomenon in milk donation.

### Study limitations

Due to the nature of qualitative research, findings presented are not intended to be representative of all Australian milk donors or breastfeeding women potentially eligible to donate in Australia. Rather, the findings provide insight into the experiences and perceptions of this cohort regarding barriers and enablers of milk donation in Australia. Further to this, women of white-Australian background (those not identifying as belonging to an ‘ethnic minority’ group in Australia; see Table 1) were overrepresented in the study sample. Culturally informed research is needed to explore the experiences and perceptions of both donors and potential donors from ethnic backgrounds considered a minority in Australia. Also, whilst heteronormative language was used for the purpose of this paper, we acknowledge that not all individuals who may be eligible to donate milk identify as women or mothers; and the potentially unique experiences of these individuals are not captured in this study.

It is also acknowledged that the experiences of donors and potential donors represent just some factors that may enable or inhibit milk donation. As noted, other factors not explored in this study may include systemic barriers (such as service operational issues) and those related to other populations involved in milk donation such as milk bank staff and healthcare professionals working with women who may be potential donors. Future research and initiatives to increase the supply of milk donors should consider these other factors.

## Conclusion

With increasing demand for milk bank services, it is imperative to have a sustainable supply of milk donors. Women who are breastfeeding or expressing milk for their own child may be unaware that donating milk is a potential option. Consideration should be given to increasing awareness of milk donation in Australia amongst both women and healthcare professionals providing services to women who may be/become milk donors. Further, to improve the recruitment and retention of milk donors, milk banks should prioritise the ease and accessibility of the service to ensure positive donor experiences.

## Supplementary Information

Below is the link to the electronic supplementary material.

Supplementary Material 1: Additional file 1: File format: pdf. Title: Semi-structured interview guide. Description: List of questions and prompts used by interviewers during participant interviews.

## Data Availability

No datasets were generated or analysed during the current study.

## Abbreviations

D: Donor
PD: Potential donor
PDHM: Pasteurised donor human milk
