# Supplementary material for: Human milk donation in Australia: a qualitative study of donors and potential donors
Source: Int Breastfeed J. 2025 Nov 18;20:87. doi: 10.1186/s13006-025-00782-w (PMC12625069; doi:10.1186/s13006-025-00782-w)
Supplement: Supplementary file 1 — Supplementary Material 1: Additional file 1: File format: pdf. Title: Semi-structured interview guide. Description: List of questions and prompts used by interviewers during participant interviews. [file 13006_2025_782_MOESM1_ESM.pdf]

Semi-structured interview guide:

Group	Topic area	Example questions/ probes
All participants	Experiences with expressing milk	Why started pumping (if same with previous babies, if applicable)<br>Pumping regimes – where, when, how long<br>What they do with stored milk (other than donate to milk bank)<br>Support from/role of family<br>Exclusivity of pumping<br>How feeding own baby<br>Did you receive any support from a health care professional with expressing/ breastfeeding? If so – type, where, when, how, why
	Perceptions of the use of donor milk	Can you tell me what you know about how donor milk is used? – how they know (i.e. told by health care professional, internet search, personal experience)<br>Have you ever had a baby in a NICU, or known someone who had a baby in a NICU – if so, can you tell me about that experience?<br>Perceptions of NICUs and their use of donated milk
Donors only	Their personal journey to becoming a donor	Number of children – and if donated with more than one child.<br>Can you tell me about your journey to becoming a milk donor?<br>How did you learn about becoming a donor? Were they informed by a health care professional – if so, type, when, where, how and why.<br>Prior knowledge of milk donation<br>Reasons for donating milk<br>Previous experiences/perceptions of milk sharing<br>The milk bank recruitment process
	Experiences and perceptions of what factors made donating milk easier or harder	Can you tell me about any challenges you experienced when donating?<br>What factors made donating easy or possible for you?<br>What could milk banks, like Lifeblood, do to make donating easier for you?
Potential-donors only	Perceptions of donating milk	Have you ever considered donating to a milk – to milk bank or milk sharing – and why?<br>Can you tell me what you know about donating to a milk bank? (why, how, where, when)<br>Has any health care professionals ever spoken to you about milk donation, if so -type, when, where, how and why.
	Experiences and perceptions of what factors made donating milk easier or harder	What factors could enable you to donate?<br>What factors may prevent/make it difficult for you to donate milk?<br>What could milk banks, like Lifeblood, do to make donating easier for you?
